# S-Nitrosoglutathione Reductase Underlies the Dysfunctional Relaxation to Nitric Oxide in Preterm Labor

**DOI:** 10.1038/s41598-018-23371-w

**Published:** 2018-04-04

**Authors:** Scott D. Barnett, Christina R. Smith, Craig C. Ulrich, Josh E. Baker, Iain L. O. Buxton

**Affiliations:** 1 Myometrial Function Laboratory, University of Nevada Reno, School of Medicine, Reno, Nevada United States; 2 Department of Pharmacology & Biochemistry, University of Nevada, Reno School of Medicine, Reno, Nevada United States

## Abstract

Tocolytics show limited efficacy to prevent preterm delivery. In uterine smooth muscle cGMP accumulation following addition of nitric oxide (NO) has little effect on relaxation suggesting a role for protein S-nitrosation. In human myometrial tissues from women in labor at term (TL), or spontaneously in labor preterm (sPTL), direct stimulation of soluble guanylyl cyclase (sGC) fails to relax myometrium, while the same treatment relaxes vascular smooth muscle completely. Unlike term myometrium, effects of NO are not only blunted in sPTL, but global protein S-nitrosation is also diminished, suggesting a dysfunctional response to NO-mediated protein S-nitrosation. Examination of the enzymatic regulator of endogenous S-nitrosoglutathione availability, S-nitrosoglutathione reductase, reveals increased expression of the reductase in preterm myometrium associated with decreased total protein S-nitrosation. Blockade of S-nitrosoglutathione reductase relaxes sPTL tissue. Addition of NO donor to the actin motility assay attenuates force. Failure of sGC activation to mediate relaxation in sPTL tissues, together with the ability of NO to relax TL, but not sPTL myometrium, suggests a unique pathway for NO-mediated relaxation in myometrium. Our results suggest that examining the action of S-nitrosation on critical contraction associated proteins central to the regulation of uterine smooth muscle contraction can reveal new tocolytic targets.

## Introduction

Approximately 15 million preterm births occur annually worldwide^1^. Preterm infants that survive are at risk for learning disabilities, cerebral palsy, vision and hearing loss, respiratory and digestive problems^2^. In 2012, more than 11% of US births were premature^3^. Although the etiology of spontaneous preterm birth is likely to be complex, disparate medical, environmental, and genetic risk factors are thought to converge on effector pathways in the uterine myometrium to influence contractility and birth timing in women^4^. Indeed, the definition of preterm labor as contractions of the uterus resulting in changes in the cervix that start before 37 weeks of completed gestation is likely to be inadequate to address the mechanism(s) of preterm labor^5^. This is in part due the probability that mechanisms underlying spontaneous preterm labor likely differ from those that are the result of infection-associated preterm birth^6^.

Tocolytics used to prevent spontaneous preterm labor (sPTL) to prevent preterm birth are not FDA approved for this purpose, and on average are said to delay labor for only 48 hours, a window for antenatal steroid, but hardly a solution to the problem. Microbial infection might initiate preterm labor (PTL) in some cases, but antibiotic treatment does not prevent preterm birth^7,8^. Because no available medications can reliably interrupt established sPTL and allow an afflicted pregnancy to continue to term, it is clear that we lack the tools needed to address this problem. Newly developed technology, such as the creation of the artificial womb^9^, can be viewed as emphasizing the severity of the problem of prematurity, as well as our lack of a complete understanding of uterine quiescence^10^.

If we are to advance our understanding of preterm birth in order to prevent it, we posit that understanding the biochemical mechanisms of relaxation of the uterus is paramount. This is because employing tools such as terbutaline, used to relax airway smooth muscle, or nifedipine, used to relax vascular smooth muscle, in an effort to prevent preterm labor are borrowed pharmacology. Even atosiban, a selective oxytocin–vasopressin receptor antagonist designed specifically to mitigate contractions of the uterus, is not approved for use in the United States and does not reduce the risk of preterm birth or improve neonatal outcome^11^. It is not unreasonable to conclude that myometrial relaxation signaling is therefore unique, and a detailed understanding of myometrial relaxation signaling is urgently needed.

Some years ago our laboratory observed the ability of nitric oxide (NO) to relax term human myometrium, and we addressed the mechanism by which this occurs^12^. While treatment of pregnant myometrium with NO donors relaxed the term tissue, blockade of cyclic GMP accumulation by inhibiting soluble guanylyl cyclase (sGC) failed to block the relaxation as predicted. These results were immediately controversial as they challenged the dogma^13^ established following the classic smooth muscle experimets of Furchgott^14^. This dogma was developed in studies of blood vessels following the discovery of an endothelial-dependent relaxing factor (EDRF) that relaxed the rabbit aorta. The currently accepted mechanism of action of NO-mediated relaxation of vascular smooth muscle posits that NO, produced by NO synthase in endothelial cells, binds to the heam moiety of soluble guanylyl cyclase in the adjacent smooth muscle to activate the smooth muscle soluble guanylyl cyclase (sGC), resulting in the accumulation of cyclic GMP in the muscle cell. Cyclic GMP then activates its cognate kinase, cyclic GMP-dependent protein kinase (PKG), leading to phosphorylation of critical contraction-associate proteins such as myosin phosphatase, and relaxation of the muscle. In myometrium, however, NO relaxes the muscle, but this is largely independent of cyclic GMP elevation^12,15^.

Our interest in NO is not aimed at proposing NO as a tocolytic. Indeed, we show here for the first time that patients who enter labor spontaneously preterm without infection have a blunted relaxation response to NO-mediated relaxation, suggesting that NO cannot be a tocolytic, and that the mechanism of NO action may be involved in the pathophysiology of preterm labor.

The biochemical distinction that relaxation of the smooth muscle of term myometrium to NO donors is cyclic GMP-independent does not argue that cyclic GMP has no role. Cyclic GMP-dependent phosphorylation can readily be measured^16^ and the ability of cyclic GMP to relax the muscle is agonist-specific since some agonists can relax the muscle in a cyclic GMP-dependent manner^17^. Moreover, the ability of NO to relax uterine smooth muscle in a fashion independent of the action of cyclic GMP is not entirely unique^18^. Nonetheless, this finding has been the subject of skeptacism despite several lines of evidence, suggesting that animal as well as human myometrium is distinct in its response to NO^12,19,20^.

Here we offer unambiguous evidence of the failure of cyclic GMP to relax myometrium and suggest that since NO relaxes term myometrium, but not preterm tissues, there is an opportunity to examine the actions of NO to identify novel mechanisms and protein targets that may be disparately regulated in preterm tissues. In addition to activation of sGC and the accumulation of cyclic GMP, NO is known to S-nitrosate proteins resulting in both redox regulation, as well as stable nitrosations, that may alter protein function^21–23^. Our findings reveal that the dysregulation of the NO modulating enzyme, S-nitrosogltathione reductase (GSNOR), in sPTL myometrium affects global protein S-nitrosation in the myometrium. The disparate protein S-nitrosations measured between term and preterm myometrium suggest that understanding the result of specific S-nitrosation differences might underly and contribute to preterm pathology. In particular, using an *in vitro* assay to investigate the interaction of the smooth muscle contractile proteins, actin and myosin, we show that protein S-nitrosation depresses cross-bridge cycling and alters acto-myosin binding dynamics consistent with relaxation of the myometrium.

## Results

### Activation SGC Fails to Relax Human Myometrium

**Figure 1:**
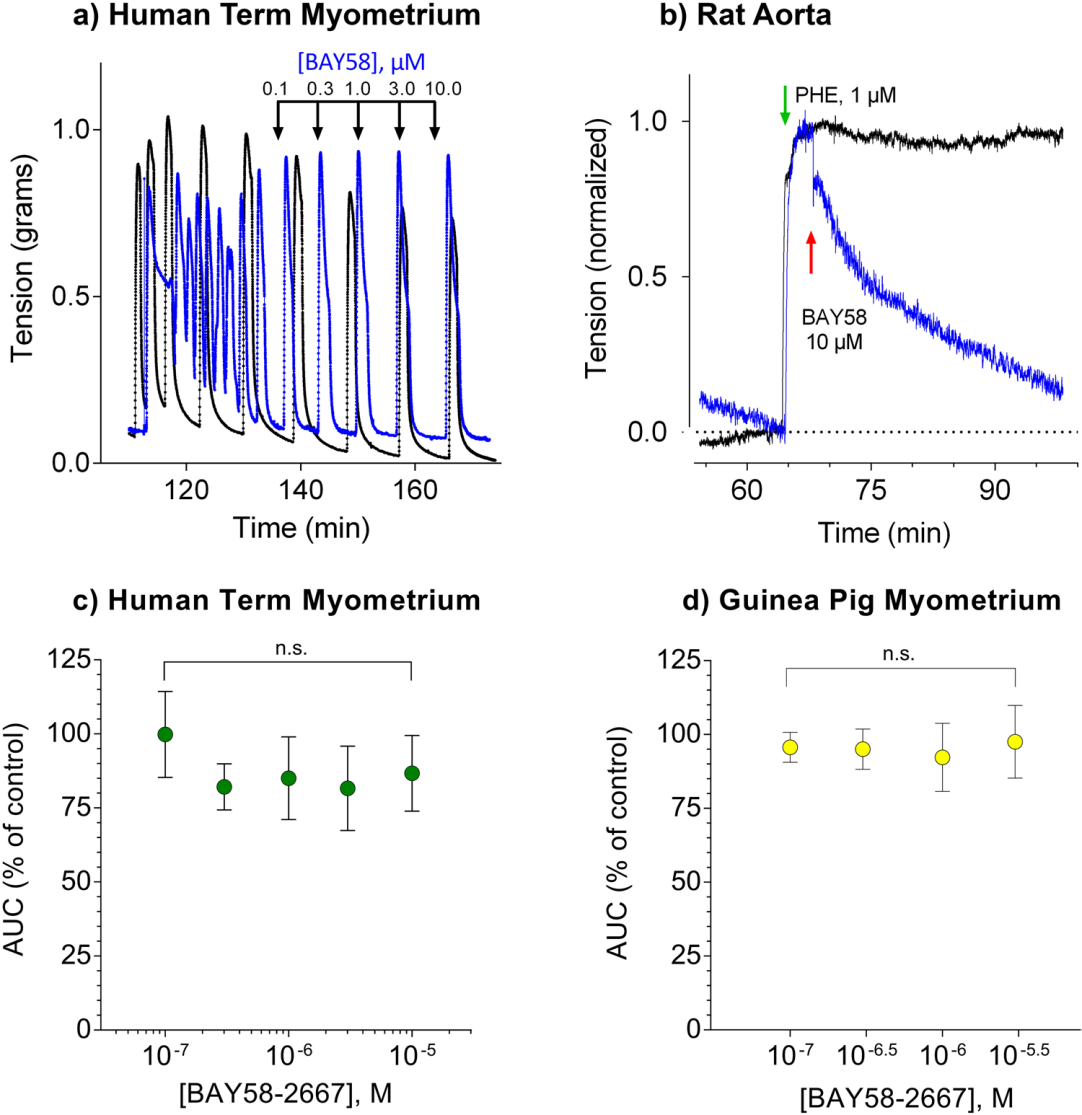
(**a**) Human pregnant term myometrium was hung in tissue baths at 37 °C with continuous oxygenation and stimulated to contract in the presence of OT. Following 60 min, tissues were challenged in the presence (blue ) or absence (black ----) of BAY58-2667 in a cumulative fashion as indicated in the figure. No response was seen to addition of BAY58-2667. Addition of the NO-donor at the end of the experiment confirmed the ability of the tissue to relax (not shown). (**b**) Rat aorta was hung in tissue baths with Krebs buffer at 37 °C with continuous oxygenation and allowed to equilibrate for 60 min in the absence of drug. Addition of phenylephrine (1 µM) produced immediate contractions (black ----) that could be relaxed (blue ) by addition of BAY58-2667 (10 µM). (**c**) The failure of tissues to relax to BAY58-2667 was repeatable in both human ( n = 4) and guinea pig ( n = 3) where control addition of GSNO at 300 μM relaxed spontaneous contractions. Data in (**c**) and (**d**) are quantified as AUC over 10 min.

In order to examine the role of cyclic GMP in uterine smooth muscle relaxation in the absence of haem-dependent activation of soluble guanylyl cyclase by NO, we have assessed the effect of direct stimulation of myometrial tissue by the haem-independent agonist BAY58-2667 (BAY58, cinaciguat) on uterine relaxation. Cinaciguat has been evaluated for haemodynamic benefit in patients with acute decompensated heart failure (ADHF)^24^. Cinaciguat is well known to stimulate cyclic GMP accumulation^25^, but fails to relax human term myometrium despite the use of concentrations from 0.1 to 10 µM administered in an increasing cumulative dose at 10 min intervals after OT stimulation (8 nM, Fig. 1a). Treatment of rat aorta with cinaciguat undertaken as a comparative control, resulted in immediate relaxation at 1 µM, consistent with the known actions of cyclic GMP in vascular smooth muscle (Fig. 1b). The EC_50_ for cinaciguat activation of the sGC is 6.4 nM and its effect to relax vascular smooth muscle is sub-μM^26^. Experiments repeated with term myometrium (Fig. 1c) from multiple human (n = 4) and guinea pig (n = 3) donors (Fig. 1d) confirmed the failure of cinaciguat to relax the tissue (one-way ANOVA: human p = 0.29; guinea pig p = 0.92).

### NO-Mediated Relaxation is Blunted in Preterm Human Myometrium

**Figure 2:**
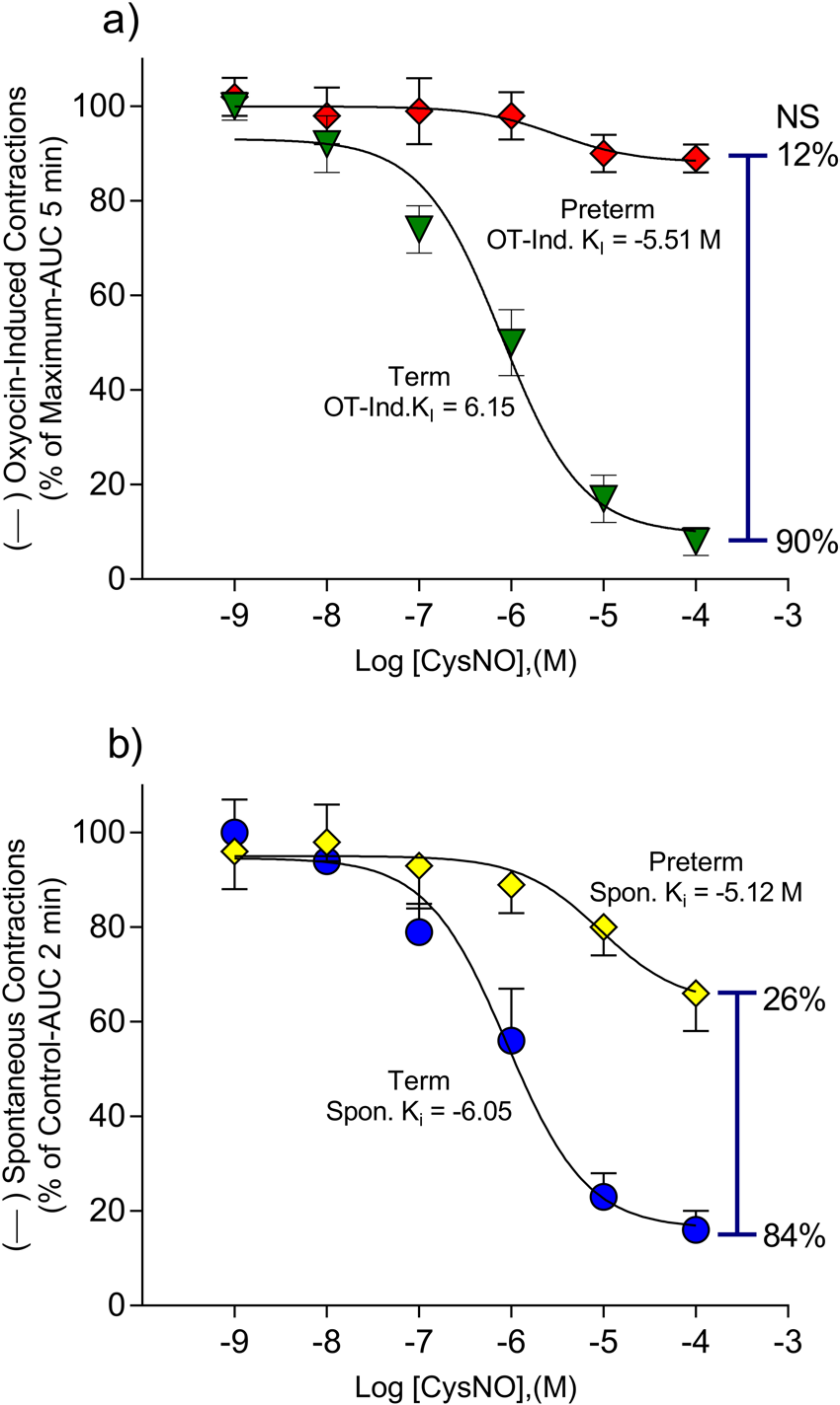
Myometrial strips from patients in labor at term or preterm were hung in tissue baths and allowed to contract spontaneously. Strips served as their own control (compared to baseline). (**a**) In tissues from women in labor at term Cys-NO relaxed OT (100 nM) induced contractions (). (**b**) In tissues from women in labor spontaneously preterm Cys-NO relaxation was insignificant () compared to relaxation in term tissue (p = 0.6). Cys-NO relaxed term laboring myometrium contracting spontaneously () while the relaxation seen in tissues from women in labor spontaneously preterm was blunted (, 26% vs. 84% relaxation) and the apparent IC50 was right-shifted 10 fold. Data are mean ± SEM of triplicate determinations in 5 patients in each pregnancy state.

Patients who enter labor spontaneously preterm without infection have a blunted relaxation response to NO (Fig. 2), suggesting that the mechanism of NO action may be involved in the pathophysiology of preterm labor. Addition of NO donor in increasing concentrations to term tissues relaxed both OT-treated (Fig. 2a) and spontaneously active tissues (Fig. 2b). Treatment of sPTL tissues in an identical fashion revealed that the NO-donor cysteine-NO (CysNO) could not relax OT-treated tissues (Fig. 2a) and the relaxation of spontaneous contractions was blunted (Fig. 2b). This is the first study to measure the ability of NO to relax spontaneous preterm *vs*. term pregnant human myometrium.

### Expression of S-Nitrosoglutathione Reductase (GSNOR) in Myometrium

**Figure 3:**
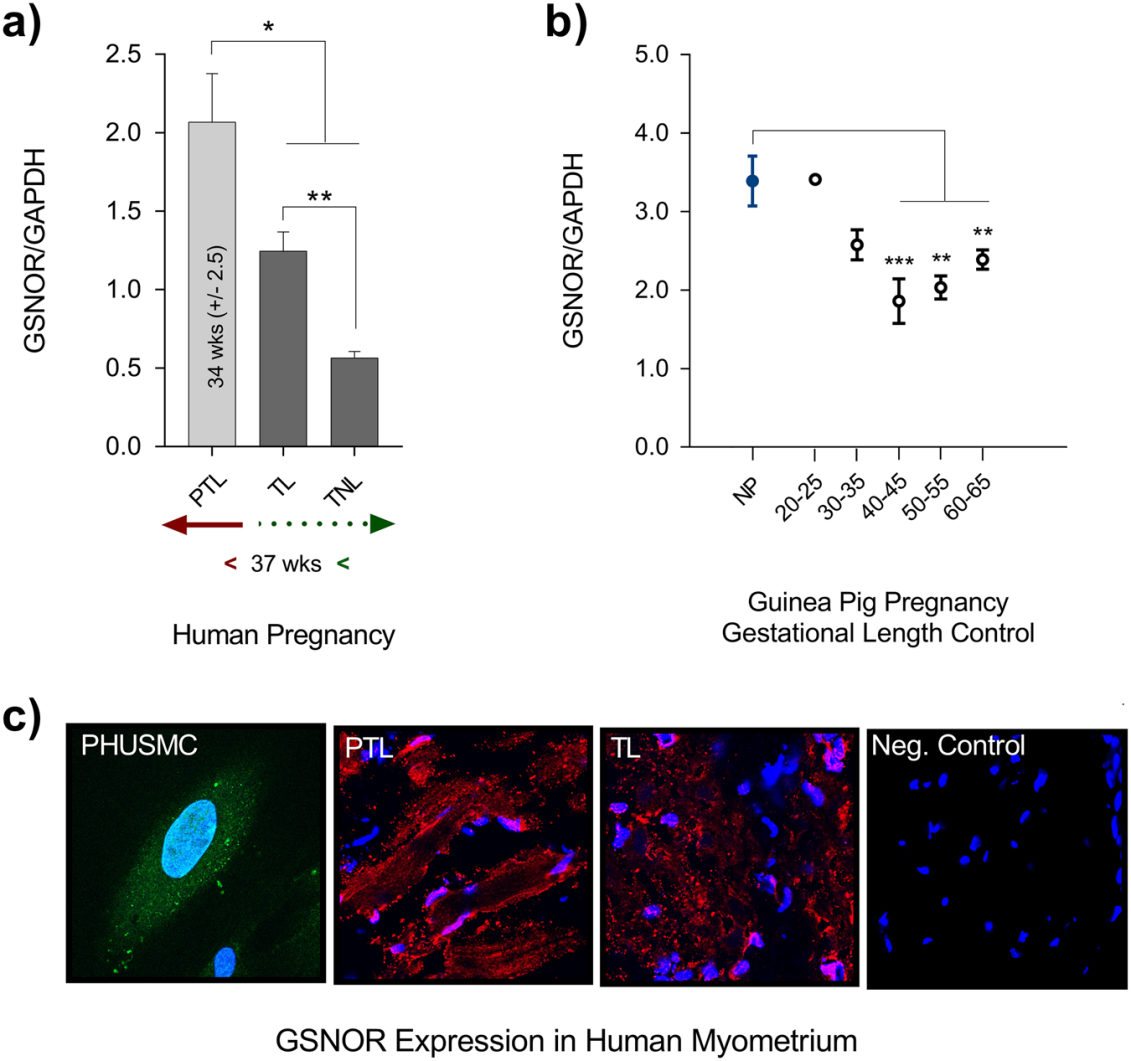
GSNOR Expression: (**a**) Wes® protein assay of normalized GSNOR expression in tissues from women laboring spontaneously preterm (PTL - mean delivery at 34 wks. ± 2.5 wks., n = 9), tissues from women in labor at term (TL, n = 8), and tissues from women non-laboring at term (TNL, n = 3). Statistical comparison by Dunnett’s multiple comparisons test; sPTL vs TL p = 0.04; PTL vs TNL p = 0.02; TL vs TNL p = 0.007. (**b**) A gestational timing control of GSNOR expression was performed in guinea pigs. GSNOR expression at several times during pregnancy was compared to non-pregnant (NP) control. (n = 3-6 at each time). Statistical comparison gestational times to NP by ANOVA (c) Confocal images of GSNOR expression in telomerized human uterine smooth muscle cells (PHUSMC 60× magnification, green = GSNOR, blue = nucleus) and whole tissue from TL, and PTL myometrial tissue (40× magnification, red = GSNOR, blue = nucleus). All data presented as mean ± SEM.

Blunted relaxation to the NO donor Cys-NO suggested the possibility that NO availability to S-nitrosate contraction associated proteins in sPTL tissue was somehow limited. Glutathione is the major thiol in mammalian cells (up to 10 mM)^27^. S-nitrosoglutathione (GSNO) is the likely form in which NO resides in the cell to trans-nitrosate proteins^28^. The metabolism of GSNO is accomplished by the Class-III alcohol dehydrogenase (ADH5)^23,29^. The principal substrate for ADH5 is GSNO. The enzyme, renamed S-nitrosoglutathione reductase (GSNOR), utilizes NADH to carry out a 2^e−^ reduction of GSNO to generate glutathione sulfinamide^30^. Using a Wes® Simple Western™ assay we determined that GSNOR protein expression increased in sPTL myometrium compared to term laboring tissue (p = 0.04) and term non-laboring (p = 0.02), and it also increased in term laboring women compared to those not in labor (p = 0.007), as determined by a Dunnett’s Multiple Comparisons test (Fig. 3a). In order to address the possibility that differences seen in sPTL were the result of gestational length timing rather than a pathological feature of sPTL, we explored GSNOR expression in guinea pig pregnancy. The pattern of GSNOR expression during gestation cannot be investigated over time in an individual pregnant patient. Because guinea pigs, like women and unlike mice, experience cyclic GMP independence of NO relaxation^19^ and do not undergo progesterone withdrawal^31^, we examined the changes in GSNOR expression during normal guinea pig gestation. GSNOR expression in guinea pigs is biphasic, dropping during gestation and returning toward pre-pregnancy levels at term (Fig. 3b), and was significantly lower than the non-pregnant (NP) guinea pig by gestational days 40–45 (p = 0.0005), 50–55 (p = 0.002), and 60–65 (p = 0.005), as determined by one-way ANOVA followed by Dunnett’s multiple comparisons test. Immunohistochemical examination of GSNOR expression in myometrial cells (PHUSMC) in culture, as well as myometrial tissue from term (TL) and preterm (sPTL) laboring patients (Fig. 3c), is consistent with measurements of protein. Decreased expression of GSNOR is expected to result in increased GSNO levels to promote critical S-nitrosations that are consistent with quiescence, while increased GSNOR expression, as with sPTL, would serve to lower the availability of GSNO and thus lower critical S-nitrosations that govern uterine quiescence.

### GSNOR Enzyme activity in Pregnant Myometrium

**Figure 4:**
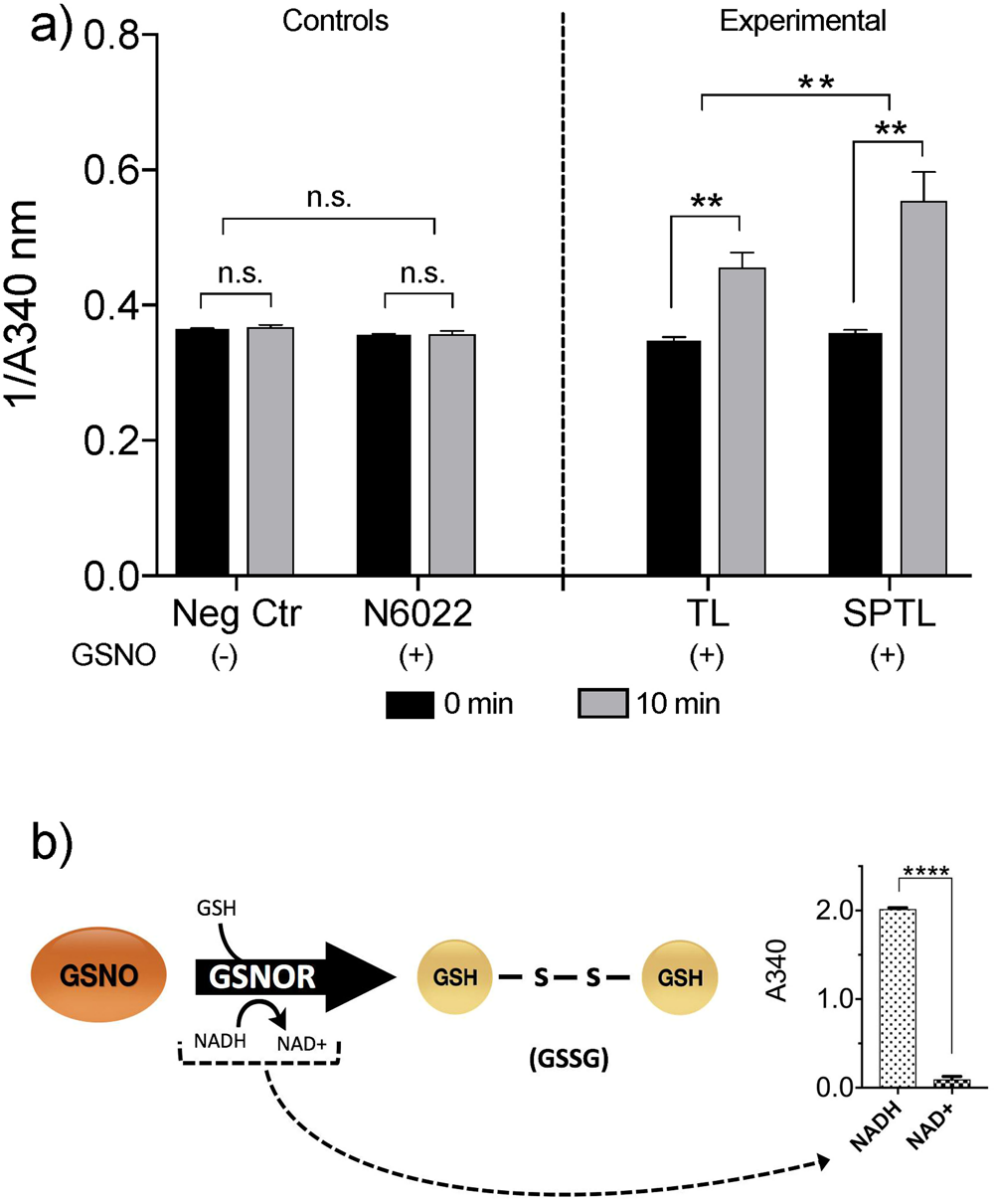
GSNOR enzyme activity assay measured at time = 0 & 10 min: (**a**) Total GSNOR activity from myometrial protein lysate (1 mg/ml) is higher in tissues from women laboring spontaneously preterm (sPTL n = 4) than in tissues from women laboring at term (TL n = 4) **p = 0.006. The addition of N6022 (8 nM) reduces GSNOR activity to baseline levels. (**b**) NADH, a required coenzyme for GSNOR activation, absorbs strongly at 340 nm. A decrease in A340nm occurs when the enzyme converts NADH to NAD+ and serves as a measure of GSNOR enzyme activity during the conversion of GSNO to glutathione disulfide (GSSG). Values (1/A340 by convention) are reported as mean ± SEM.

Increased protein expression of GSNOR suggested the possibility that enzyme activity would also be increased. We adapted an assay that followed NADH consumption in an GSNOR specific fashion^32^ (Fig. 4). N6022 (3-(5-(4-(1H-imidazol-1-yl) phenyl)−1-(4-carbamoyl- 2-methylphenyl)−1H-pyrrol-2-yl) propionic acid) from Nivalis Pharmaceuticals, is known to be a potent and specific inhibitor of GSNOR^33^. GSNOR activity is elevated in sPTL (Fig. 4a) compared to TL (two-tailed, unpaired student’s t-test p = 0.006) consistent with the notion that lowered GSNO levels contribute to the failure of NO to relax sPTL tissues and may play a role in the pathophysiology of spontaneous preterm labor. The specificity of the assay (Fig. 4b) is demonstrated by the complete reliance on NADH.

### Blockade of GSNOR Activity Relaxes Term Non-Laboring (TNL) Myometrium

**Figure 5:**
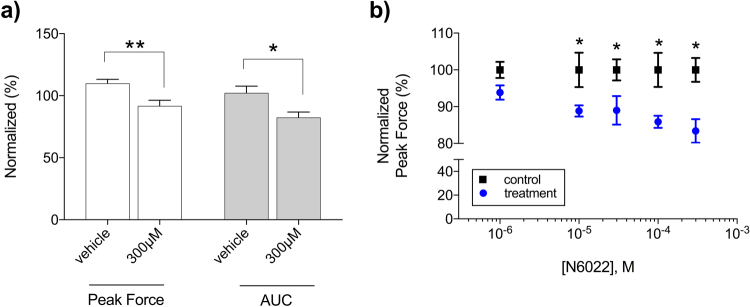
Myometrial Response to GSNOR inhibition. (**a**) 300 µM N6022 was applied to myometrial tissue strips from women in not in labor at term (TNL, n = 3) in an organ bath for 30 min. Peak force and area under the curve (AUC) analyzed as compared to a vehicle control (DMSO). (**b**) An increasing cumulative dose of N6022 (, 1µM-300µM) was added to a bath with TNL myometrial tissue strips (n = 3) at 15 min intervals. All data presented as mean ± SEM.

In order to determine if GSNOR actively contributes to myometrial quiescence, we inhibited the enzyme with N6022, a potent and selective inhibitor of GSNOR. *Ex vivo* organ bath experiments using TNL myometrium revealed that the addition of N6022 relaxes the term non-laboring myometrium (Fig. 5a) whether quantified as peak force or area under the curve (AUC) that represents force over time. The effect of GSNOR inhibition is dose-dependent (Fig. 5b). While the effect of N6022 is modest (~15–20% relaxation), it is notable that GSNOR inhibition affects force, and that force reduction may be consistent with S-nitrosations lowering the threshold for contractions until term. Reduction in both peak (p = 0.005) force and area under the curve (p = 0.013) is consistent with S-nitrosation differences that favor relaxation.

### The Effect of GSNO on Protein S-Nitrosation in Human Myometrium

**Figure 6:**
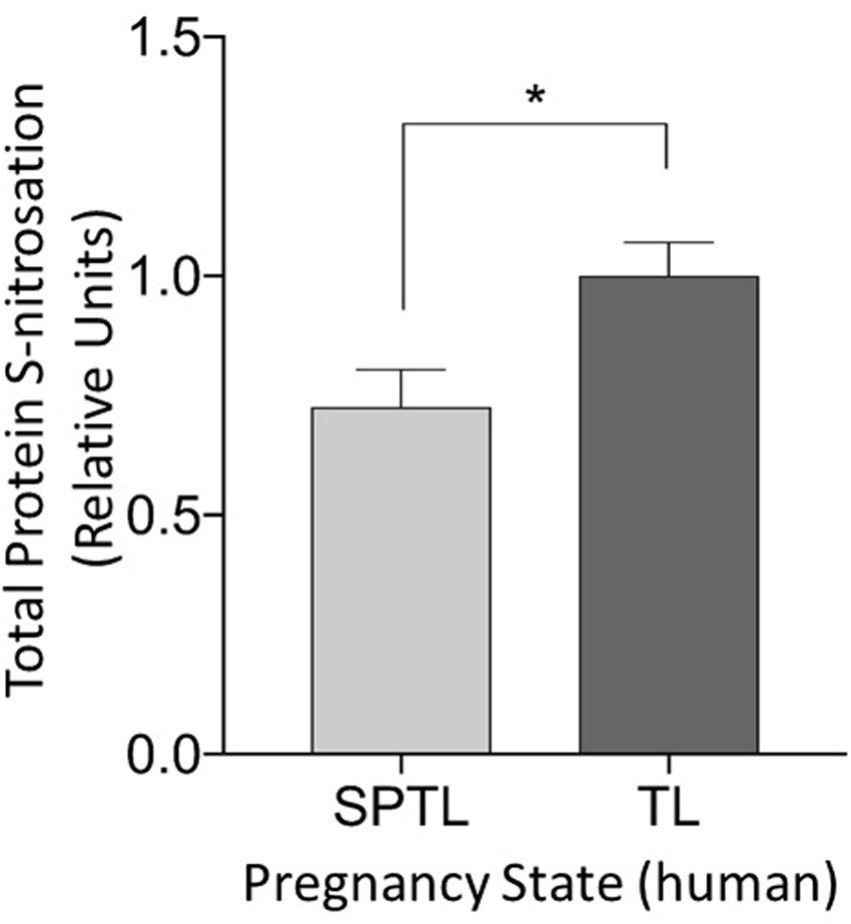
Relative expression of human uterine smooth muscle S-nitrosated proteins in tissues from sPTL patients (n = 12) versus patients in labor at term (n = 12) when isolated myometrium is treated with GSNO. Data represent a compilation of total protein S-nitrosations in each tissue state controlled for total protein abundance. S-nitrosations are significantly lower in sPTL tissues (p < 0.05).

Total protein S-nitrosations were measured in GSNO treated term and sPTL myometrium using LC/MS/MS. S-nitrosated peptides were quantified and expression levels of 110 S-nitrosated proteins were quantified using normalized spectral counts and AUC measures of extracted ion chromatograms. We employed both quantification measures because spectral counting, while using dynamic exclusion, limits the quantitative capability to a relative measurement between states and we wanted to verify that the number of MS2 events per peptide were indicative of the amount of peptide that was measured by AUC of the MS1 chromatograms. ANOVA demonstrated that proteins exhibited statistically significant differences between TL and sPTL tissues (Fig. 6) specified by an F statistic P value of P < 0.05 (Fig. 6). These proteins had log_2_ fold changes of at least ±1 in preterm laboring patients compared with term laboring patients. Stringent controls were performed to avoid false positive identification of non-S-nitrosated cysteines that could have been mislabeled during the experimental procedures. These controls are standard when performing the biotin switch procedure and include removal of GSNO and/or ascorbate during the biotin switch. A small number of proteins were shown to be constitutively S-nitrosated and were labeled without the addition of GSNO. This is a common and expected result. Removal of ascorbate during the biotin switch removed any signal that was seen when ascorbate was present. Streptavidin purification of biotin switched proteins removes any signal from naturally biotinylated proteins, and these are therefore not present in our analysis. Streptavidin purification and LC/MS/MS analysis of ascorbate negative samples were shown to only contain the contaminating keratin proteins, trypsin and traces of serum albumin. Therefore, our ascorbate-positive sample identification contains almost no nonspecific binding proteins.

### GSNO Alters Actin-Myosin Motility

**Figure 7:**
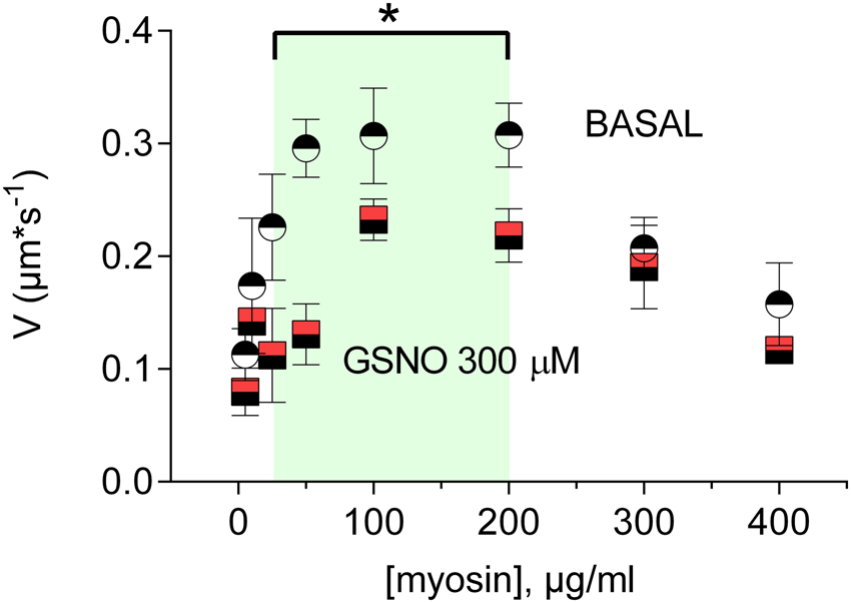
Actin Motility Assay: Actin velocities (±300 µM GSNO) reveal changes in ATPase activity and acto-myosin binding kinetics. Phosphorylated uterine smooth muscle myosin (pSMM) binds to a nitrocellulose-coated cover slip and is inverted onto a glass slide to form a flow cell. TRITC-labeled actin (10 nM) and ATP (1 mM) ± GSNO are combined in the flow-cell and actin velocities are measured using video imaging. All data presented as mean ± SEM.

In order to examine the functional effect of protein S-nitrosation in human myometrium, myosin-dependent actin dynamics were measured as translocation, or sliding, of actin filaments over smooth muscle myosin bound to a coverslip. The assay permits determination of the velocity of the interaction as a measure of cross-bridge cycling. Addition of GSNO to the motility assay results in a reduction in velocity consistent with an effect of GSNO to S-nitrosate one or more constituents in the assay, specifically smooth muscle myosin, and/or actin, both of which contain numerous cysteines (Fig. 7). At myosin densities of 25, 50, 100, 200, and 400 (µg/ml), the addition of 300 µM GSNO significantly decreased actin velocity P < 0.05. The result is consistent with an effect of GSNO to relax the smooth muscle and suggests a mechanism for S-nitrosation of contraction associated proteins underling the non-cyclic GMP action of NO.

## Discussion

Despite decades of research, tocolytics do not reliably extend pregnancy beyond 48 hours. Approaches to manage sPTL are employed without clear evidence of benefit for acute or maintenance tocolysis^34,35^, and no tocolytic currently in use is without potential adverse effects^36^. It is imperative that new approaches to prevent preterm contractions of the uterus be found. An intriguing observation in uterine smooth muscle is its ability to relax independently of sGC-mediated cyclic GMP accumulation when exposed to NO^12,19,20,37^. Here we address three questions: (1) How does sPTL myometrium respond to NO; (2) what alternative pathways might NO act upon to mediate relaxation in the myometrium; and (3), is it possible that the dysregulation of GSNOR, an important modulator of S-nitrosations in the myometrium, is responsible for the decreased level of protein S-nitrosation measured in sPTL tissues, and does GSNOR serve as a contributing factor to the blunted relaxation response to NO in spontaneous preterm labor?

Our lab has previously determined that the inhibition of sGC by agents that prevent cyclic GMP accumulation do not prevent NO-mediated relaxation of the human term myometrium^15^. Soluble GC converts GTP to cyclic GMP to activate its cognate kinase, PKG, whose targets include myosin phosphatase, which dephosphorylates the regulatory light chain^38^. We do not posit that cyclic GMP has no role in myometrium, indeed, we have shown that accumulation of cyclic GMP following activation of the particulate guanylyl cyclase does relax the uterus^17^. These results suggest compartmentation of second messenger signaling in myometrium, a finding first established over three decades ago in cardiac myocytes^39^. This signaling exception in myometrium represents a unique opportunity to discover a new approach to tocolytic development. The failure of NO-induced sGC-mediated cyclic GMP accumulation to relax myometrial smooth muscle was unexpected. The assumption that cyclic GMP measurements are sufficient to make the case is refuted by evidence establishing that blockade of cyclic GMP accumulation does not prevent the relaxation. Direct evidence for the failure of cyclic GMP to signal myometrial quiescence is further established by our finding that the activation of sGC by the haem-independent agonist cinaciguat does not promote relaxation of the myometrium (Fig. 1a,c,d), despite its robust capability to do so in other smooth muscles (Fig. 1b). We propose that NO drives an alternative pathway in myometrial smooth muscle integral to contraction/relaxation cycling.

One such pathway that may impact smooth muscle relaxation is protein S-nitrosation. Not only is this posttranslational modification important to cellular homeostasis, its dysregulation can drive disease states^23^ and smooth muscle inflammation^40,41^. It is generally accepted that pregnancy is, to some degree, an inflammatory condition, whether due to maternal infection or not^42,43^, and we suggest that dysregulation of S-nitrosation of critical contractile proteins underlies sPTL both because S-nitrosation impacts contractile protein regulation as we show, and because S-nitrosation is a principal redox regulator^44^ that may limit inflammatory mechanisms until term.

We have shown that many proteins critical to the uterine contraction/relaxation cycle are differentially S-nitrosated in in preterm laboring myometrium as compared to term laboring, and term non-laboring, tissue^45^. We investigated the functional relevance of protein S-nitrosation by examining the effects of GSNO addition on ATP-ase activity and acto/myosin binding dynamics (Fig. 7). The presence of GSNO in the actin motility assay depresses ATP-ase activity, and by extension, cross-bridge cycling. Furthermore, as suggested by a decrease in the slope of actin velocity at low myosin concentrations, we can infer that the acto-myosin “latch phase” time is likely lengthened. The notion that S-nitrosation can impact contractile mechanisms has been suggested by studies in skeletal muscle^46,47^. Both of these contributions were made examining skeletal muscle, which differs markedly from uterine smooth muscle. The motility assay data presented here are compelling because they employ purified smooth muscle myosin, and because they support an effect of GSNO to promote relaxation through S-nitrosation in the absence of cyclic GMP elevation.

Protein S-nitrosation is an important regulator to human health and disease^48^, and the level of S-nitrosation in the cell is highly dependent on the availability of intracellular GSNO^49^. As GSNO concentrations increase, so do levels of total protein S-nitrosation. Because GSNOR (^−/−^) mice have increased cellular levels of GSNO and SNO-proteins^50^, it is likely that GSNO and some S-NO-proteins are in equilibrium governed by Cys-to-Cys trans-nitrosation, and GSNOR de-nitrosation. We have found that women who undergo sPTL show decreased protein S-nitrosation (Fig. 6), and exhibit higher cytosolic levels and activity of GSNOR (Figs 3, 4), an enzyme that metabolizes GSNO to GSSG (Fig. 4b). Taken together with our finding that women who undergo sPTL present a blunted response to NO-mediated relaxation (Fig. 2), we conclude that GSNOR may be responsible for atypical myometrial activity in some women who undergo sPTL. We do not posit that the dysfunction of sPTL tissues to NO is a matter of availability *per se*. Indeed, no concentration of NO donor overcomes the dysfunction we demonstrate. What is likely is that some specific S-nitrosation differences are the result of regulation by GSNO metabolism. This conclusion is supported by or demonstration of both up and down S-nitrosation difference seen in sPTL tissues^45^.

The knowledge that GSNOR regulates GSNO availability, and that GSNOR is upregulated in sPTL myometrium, makes it an attractive therapeutic target. N6022, a potent and selective inhibitor of GSNOR, is well tolerated in humans and has already been tested in clinical trials as an airway smooth muscle relaxing agent in asthmatics, and as a treatment for cystic fibrosis^51^. Here we demonstrate its efficacy, and entertain its use as a tocolytic (Fig. 5). In organ bath experiments N6022 significantly decreased the peak force of contraction and AUC of term myometrium. Due to the drug’s modest bioavailability (~10%)^52^, its potential as a marketable tocolytic remains in question. However, numerous derivatives of N6022 have been identified^53^ as well as other compounds targeting GSNOR^40^ that may prove useful as tocolytic agents. Beyond GSNOR, there are other NO-mediators in the cell such as the thioredoxin system^54^ and carbonyl reductase^27^ that may also prove attractive as therapeutic targets to increase the availability of critical protein S-nitrosations in the cell.

Ultimately, the underlying cause(s) of sPTL remain unknown. Here we provide a novel mechanism that may provide insight into the unique relaxation pathway in the myometrium. Our finding that GSNOR, and by extension protein S-nitrosation, are dysregulated in the myometrium of women undergoing sPTL affords an opportunity to investigate a new class of drugs as tocolytics.

## Methods

### Tissue collection

Human tissue collection and research was approved by the University of Nevada Biomedical Review Committee for the protection of human subjects. Human uterine biopsies were obtained with written informed-consent from mothers with singleton pregnancies undergoing Cesarean section without known infection or rupture of membranes. All experiments were performed in accordance with the NIH guidance on the use of human tissues in research. The absence of maternal infection was determined by the obstetrical team at the time of surgery based on the appearance of placenta and membranes. The possibility of subclinical infection in patients delivering preterm is not ruled out. Exclusion criteria include age < 18 years, any history of drug abuse, co-morbid diagnoses such as HIV infection or AIDS, hepatitis C infection, uncontrolled diabetes, renal disease, preeclampsia, IUGR, placenta previa and any use of steroids other than betamethasone including topical use during pregnancy. Tissues were transported to the laboratory immediately in cold Krebs buffer containing: NaCl (118 mM), KCl (4.75 mM), CaCl_2_ (2.5 mM), KH_2_PO_4_ (1.2 mM), NaHCO_3_ (25 mM), MgCl_2_ (1.2 mM), dextrose (20 mM), and adjusted to pH 7.4. Tissues were microdissected under magnification to isolate smooth muscle, employed in contractile experiments or snap frozen in liquid nitrogen, and stored at -150 °C. The average age for patients in the pregnant laboring group was 28.9 ± 5.6 yr. and in the preterm laboring group 30.8 ± 10.2 yr. Pregnant laboring patients ranged from 39 to 41 wk. gestation, with the mean at 39 wk. Preterm laboring patients without evidence of infection, PROM or preeclampsia ranged from 29.2 to 36 wk. of gestation, with the mean being 33.5 wk. All preterm patients delivered male fetuses. Patients recruited for our experiments were Caucasian or Hispanic.

Animal studies were approved by the University Institutional Animal Care and Use Committee. All experiments were conducted in accordance with the NIH guidelines for the use of vertebrate animals in research. Dunkin-Hartley Guinea pigs (Elm Hill, Chelmsford, MA) were purchased as either virgin juveniles (300–350 g) and bred on site, or as timed-pregnancies (30–35d). Non-pregnant guinea pigs were estrogen primed (3 mg/kg β-estradiol) 48-hours prior to tissue collection to ensure alignment of estrous cycles. Virgin female guinea pigs, and timed-pregnant animals, were sacrificed under isoflurane anesthesia. Uterine tissue was dissected and used immediately as previously described^55^.

### Contractile studies

Strips of myometrium (~0.5 × 15 mM) were clip-mounted by silk thread, attached to a force transducer, and isometrically stretched to an initial tension of 1.2 × tissue length in an organ bath (WPI, Sarasota, FL) containing Krebs buffer. Tissues were maintained at 37 °C and gently bubbled with balanced oxygen (95% O_2_, 5% CO_2_). Tissues were then challenged with KCL (60 mM replacing NaCl) for 3 min, followed by washout, then allowed to equilibrate for 1 hr. during which time regular spontaneous contractions were seen. Only tissues that responded to KCL-challenge were employed in experiments. Under some conditions, tissues were further challenged with oxytocin (OT, 8 or 100 nM), followed by washout. Tissues served as their own controls based on tension prior to addition of drug. Both NO donors Cysteine-NO (100 µM) or GSNO (300 µM) were made daily. Data were analyzed with LabScribe (version 3.015800, Mac OS 10.11, iWorx systems Inc., Dover, NH). Aorta was collected from 3-month-old Sprague-Dawley rats, cut into 2 mm rings, and hung by stainless steel triangles that were passed through the lumen of each ring.

### Wes Protein Assay

Each sample was ground to a powder under liquid nitrogen and reconstituted in RIPA buffer (0.8 mg/ml final): Tris pH 7.5 (20 mM), NaCl (150 mM), EDTA (1 mM), EGTA (1 mM), NP-40 (1%), sodium deoxycholate (1%), and protease inhibitors (cat.78430: Thermo Fisher Scientific Inc., Waltham, MA). Wes was run according to manufacturer protocols (SM-W004 - ProteinSimple, San Jose, CA) using a 12–230 kDa Wes Separation Module coupled to a 25-capillary cartridge. The Wes system is not a typical immunoblot method where qualitative images are quantified to generate data, rather, chemiluminescence is detected and quantified directly. GSNOR was labeled with rabbit anti-ADH5 primary antibody (1:100 dilution, ab59134: Abcam, Cambridge, MA) and mouse anti-GAPDH (1:100 dilution, sc-47724: Santa Cruz Biotechnology, Inc., Dallas, TX). GSNOR and GAPDH were not multiplexed due to insufficient separation of bands as a result of similar molecular weights. Protein identification and quantification was determined using Compass software (version: 2.7.1, Mac OS 10.11: ProteinSimple, San Jose, CA), followed by Prism (version 7.0c for Mac OS 10.11, GraphPad Software, La Jolla California USA), when necessary.

### Enzyme Activity Assay

The GSNOR activity assay was performed as previously described^29^ using total protein lysate from human uterine smooth muscle tissue taken from the superior portion of the transverse incision. The lysate was prepared to a final concentration of 1 mg/ml in oxygen-purged experimental buffer containing: Tris-HCl pH 8.0 (20 mM), EDTA (0.5 mM), NP-40 (0.1%) and 1 mM phenylmethylsulphonyl fluoride (PMSF). Lysate was equilibrated at r.t. for 10 min in the presence of NADH (300 µM) prior to addition of GSNO (200 µM). Absorbance at 340 nm (A340) (SmartSpec^TM^ Plus: Bio-Rad Laboratories, Inc., Hercules, CA) was recorded at t = 0, 5, 10 min to ensure stability of the NADH pool prior to the addition of GSNOR and/or inhibitors. Following equilibration, GSNO was added to the lysate mixture and A340 recording were collected at time = 0, 5, 10 min. N6022 (8 nM) (S77589: Selleck Chemicals, Houston, TX), a GSNOR inhibitor, was used to verify negligible NADH conversion to NAD^+^ in the presence of GSNO. For control experiments N6022 was added concurrently with NADH to the protein lysate and equilibrated for 10 min prior to the addition of GSNO. Absorbance data are presented as 1/A340 to represent GSNOR-dependent NADH consumption.

### Confocal Microscopy

Myometrial tissues were sliced into 10μm sections using a cryostat at −30 °C and placed on coated slides (Surgipath, Buffalo Grove, Illinois). Samples were fixed in 4% paraformaldehyde then permeabilized with 0.5% Triton X-100. GSNOR protein was labeled with goat anti-rabbit ADH5 (1:250) primary antibody (ab59134: Abcam, Cambridge, MA), followed by either TRITC-donkey anti-rabbit secondary (Santa Cruz Biotech) for tissue sections, or FITC- donkey anti-rabbit secondary (Santa Cruz Biotech) for cultured cells, then mounted in Vectashield plus DAPI (VectorLabs, Burlingame, California). Images were acquired on an Olympus IX81 Fluoview confocal microscope system at 40 × magnification and analyzed with bundled software FV10-ASW (version 04.02, Windows 7 professional, Olympus America, Inc., Melville, NY). Brightness and contrast were the only modifications to the images, and they were adjusted globally using identical values for each image to ensure consistency (brightness + 256, contrast + 53, Photoshop CC 2017.1.0, Adobe Systems Inc., San Jose, CA).

### Phosphorylated smooth muscle myosin (pSMM) preparation

SMM was isolated from frozen chicken gizzard (ID: 43018–2: Pel-Freez Biologicals, Rogers, AR) as previously described^56^. The resulting SMM product was dialyzed twice for 8 hours each in 2-liters of DTT-free HMM buffer, described below, using a 3 ml, 3.5 kDa dialysis cassette (66330: Thermo Fisher Scientific Inc., Waltham, MA). Phosphorylation of the regulatory light chain (MYL9) was also performed as previously described^57^ in DTT-free HMM buffer: MOPS (10 mM), EGTA (0.2 mM), NaCl (50 mM), CaCl_2_ (3 mM), MgCl_2_ (2 mM), and ATP (1 mM) (#A3377: Sigma-Aldrich, St. Louis, MO).

### Actin Motility Assay

The actin motility assay was performed as previously described^58^ with minor modifications to minimize the presence of DTT in the flow chamber. Flow cells were prepared by flushing the following reagents through the flow chamber and incubating at each step for one min prior to the next addition: i) myosin added in the appropriate experimental concentration (5,10, 25, 50, 100, 200, 300, 400 µg/ml), ii) BSA (5 mg/mL), iii) TRITC-labeled actin (10 nM), iv) two washes of DTT-free actin buffer (with or without 300 µM GSNO), and v) two washes of DTT-free motility buffer containing 1 mM ATP (with or without 300 µM GSNO). Motility assays were implemented using a Nikon TE2000 epifluorescence microscope with fluorescent images digitally acquired with an Andor iXon Ultra (Belfast, N. Ireland) camera. Each flow cell was imaged for 30 sec from three distinct fields to obtain a single sample (n = 1), and performed in triplicate. Data were analyzed using Simple PCI tracking software (Compix, Sewickley, PA) to obtain actin-sliding velocities. Objects were defined by applying an exclusionary area threshold to minimize background noise. Velocities too slow to be accurately measured by the PCI tracking software were hand-calculated in ImageJ (version 1.50i, Mac OS 10.11) by recording the linear velocity of (3) filaments per recording (9 velocities per n = 1). The action of GSNO was controlled by addition of GSH which had no effect.

### Protein Isolation for S-nitrosation Measurement

Myometrial muscle samples were collected from 12 patients in each pregnancy state. Tissues were ground to a powder under liquid nitrogen and reconstituted in 20 ml HEN buffer: HEPES-NaOH (25 mM), EDTA (1 mM), and neocuproine (0.1 mM, pH 7.7). Samples were sonicated (10 × 2-sed bursts, 70% duty cycle) and brought to CHAPS (0.4%) (3-(3-cholamidopropyl)dimethylammonio-1-propanesulfonate). Samples were centrifuged at 2,000 × g for 10 min at 4 °C. Protein concentration was determined by the bicinchoninic acid assay and samples diluted to 0.8 mg/ml in HEN buffer.

### Biotin Switch and Streptavidin Pulldown

Samples from each patient in each group was independently isolated by biotin switch and streptavidin pulldown and then pooled for tandem mass spectrometry (MS/MS) analysis (i.e., sPTL1, 4 unique patients; sPTL2, 4 unique patients; sPTL3, 4 unique patients; for a total of 12 unique patients split into 3 biological replicates to help control for human diversity). Pooling patients allows experimental economy while maintaining replication. Protein isolates (1.8 ml 0.8 mg/ml in HEN buffer) were incubated with GSNO (300 μM) for 20 min at r.t. At this concentration, GSNO will produce ~5μM reactive NO over 15–20 min without accumulation^59^. This reactive species concentration matches the IC_50_ concentration for relaxation of isolated myometrium^37^. Neither biotin-HPDP nor a maleimide dye lead to false positives because the amines or tyrosines are not be labeled even if nitrosated. SDS (0.2 ml of 25% SDS) was added along with 30 mM NEM. Samples were incubated at 50 °C in the dark for 20 min and proteins precipitated in −20 °C acetone for 1 hr. and collected by centrifugation at 3,000 g for 10 min. The clear supernatant was aspirated, and the protein pellet was gently washed with 70% acetone (4 × 5 ml). After resuspension in 0.24 ml HEN buffer with 1% SDS (HENS), the material was transferred to a fresh 1.7-ml microfuge tube containing 30 μl biotin-HPDP (2.5 mg/ml). The labeling reaction was initiated by addition of 30 μl of 200 mM sodium ascorbate (final 20 mM ascorbate) for 1 hr. at r.t. in the dark. Four volumes of −20 °C acetone were added to the labeled samples and incubated at −20 °C for 20 min to remove biotin-HPDP. The samples were centrifuged at 3,000 g for 10 min at 4 °C, and the supernatant discarded. The pellet was resuspended in 140 μl of HENS buffer. Neutralization buffer (HEPES (20 mM) pH 7.7, NaCl (100 mM), EDTA (1 mM), and Triton X-100 (0.5%)), was added (280 μl) along with 42 μl of streptavidin-agarose. Proteins were incubated for 1 hr. at r.t. and washed five times with 1.5 ml of neutralization buffer with 600 mM NaCl. Beads were incubated with 100 μl elution buffer (neutralization buffer with 600 mM NaCl plus 100 mM β-mercaptoethanol) to recover the bound proteins. This step releases the protein from the streptavidin bead leaving the biotin-HPDP tag bound to the bead as well as natively biotinylated proteins still bound to the bead. Four volumes of −20 °C acetone were added to re-precipitate proteins. Samples were centrifuged at 3,000 g for 10 min at 4 °C, the supernatant was discarded, and the pellet was washed and dried for proteomic analysis.

### Mass Spectrometry

The Nevada Proteomics Center analyzed proteins by trypsin digestion and liquid chromatography (LC)/MS/MS analysis. Acetone-precipitated pellets were washed twice with 25 mM ammonium bicarbonate and 100% acetonitrile, reduced, and alkylated using 10 mM dithiothreitol and 100 mM iodoacetamide and incubated with 75 ng sequencing grade modified porcine trypsin (Promega, Fitchburg WI) in 25 mM ammonium bicarbonate overnight at 37 °C. Peptides were first separated by Michrom Paradigm Multi-Dimensional Liquid Chromatography (MDLC) instrument [Magic C18AQ 3μ 200 Å (0.2 × 50 mM) column (Michrom Bioresources, Auburn, CA) with an Agilent ZORBAX 300SB-C18 5μ (5 × 0.3 mM) trap (Agilent Technologies, Santa Clara, CA)] using a 0.1% formic acid/1% formic acid in acetonitrile gradient. Eluted peptides were analyzed using a Thermo Finnigan LTQOrbitrap using Xcalibur v 2.0.7. MS spectra (m/z 300–2,000) were acquired in the positive ion mode with resolution of 60,000 in profile mode. The top 4 data-dependent signals were analyzed by MS/MS with CID activation, minimum signal of 50,000, isolation width of 3.0, and normalized collision energy of 35.0 with a targeted reject list^45^. Dynamic exclusion settings were used with a repeat count of two, repeat duration of 10 s, exclusion list size of 500, and exclusion duration of 30 s.

### Criteria for S-Nitrosation Identification

PROTEOIQ (V2.6, www.nusep.com) was used to validate MS/MS-based peptide and protein identifications. Peptides were parsed before analysis with a minimum Xcorr value of 1.5 and a minimum length of six amino acids. There were no matches to the concatenated decoy database, and therefore, a false discovery value is not applicable or “0.” Peptide identifications were accepted if they could be established at > 95.0% probability as specified by the Peptide Prophet algorithm^60^. Protein identifications were accepted if they could be established at > 95.0% probability and contained at least two identified peptides with five spectra per peptide. Protein probabilities were assigned by the Protein Prophet algorithm^61^. Proteins that contained similar peptides and could not be differentiated based on MS/MS analysis alone were grouped to satisfy the principles of parsimony.

### Data availability

The datasets generated during and/or analyzed during the current study are available from the corresponding author on reasonable request.
